# The Efficacity of the NeuroAssist Robotic System for Motor Rehabilitation of the Upper Limb—Promising Results from a Pilot Study

**DOI:** 10.3390/jcm12020425

**Published:** 2023-01-04

**Authors:** Nicoleta Tohanean, Paul Tucan, Oana-Maria Vanta, Cristian Abrudan, Sebastian Pintea, Bogdan Gherman, Alin Burz, Alexandru Banica, Calin Vaida, Deborah Alice Neguran, Andreea Ordog, Daniela Tarnita, Doina Pisla

**Affiliations:** 1 Neurology I Department, Cluj-Napoca Emergency Clinical County Hospital, 400012 Cluj-Napoca, Romania; 2 Neurology Department, University of Medicine and Pharmacy “Iuliu Hatieganu”, 400012 Cluj-Napoca, Romania; 3 CESTER, Research Center for Industrial Robots Simulation and Testing, Technical University of Cluj-Napoca, 400641 Cluj-Napoca, Romania; 4 Neurosurgery Department, Cluj-Napoca Emergency Clinical County Hospital, 400349 Cluj-Napoca, Romania; 5 Department of Psychology, Babes-Bolyai University, 400029 Cluj-Napoca, Romania; 6 Faculty of Mechanics, University of Craiova, 200512 Craiova, Romania

**Keywords:** upper limb rehabilitation, NeuroAssist system, neurorobotics, robot-assisted therapies, electromyography, motor recovery

## Abstract

The research aimed to evaluate the efficacy of the NeuroAssist, a parallel robotic system comprised of three robotic modules equipped with human–robot interaction capabilities, an internal sensor system for torque monitoring, and an external sensor system for real-time patient monitoring for the motor rehabilitation of the shoulder, elbow, and wrist. The study enrolled 10 consecutive patients with right upper limb paresis caused by stroke, traumatic spinal cord disease, or multiple sclerosis admitted to the Neurology I Department of Cluj-Napoca Emergency County Hospital. The patients were evaluated clinically and electrophysiologically before (T1) and after the intervention (T2). The intervention consisted of five consecutive daily sessions of 30–45 min each of 30 passive repetitive movements performed with the robot. There were significant differences (Wilcoxon signed-rank test) between baseline and end-point clinical parameters, specifically for the Barthel Index (53.00 ± 37.72 vs. 60.50 ± 36.39, *p* = 0.016) and Activities of Daily Living Index (4.70 ± 3.43 vs. 5.50 ± 3.80, *p* = 0.038). The goniometric parameters improved: shoulder flexion (70.00 ± 56.61 vs. 80.00 ± 63.59, *p* = 0.026); wrist flexion/extension (34.00 ± 28.75 vs. 42.50 ± 33.7, *p* = 0.042)/(30.00 ± 22.97 vs. 41.00 ± 30.62, *p* = 0.042); ulnar deviation (23.50 ± 19.44 vs. 33.50 ± 24.15, *p* = 0.027); and radial deviation (17.50 ± 18.14 vs. 27.00 ± 24.85, *p* = 0.027). There was a difference in muscle activation of the extensor digitorum communis muscle (1.00 ± 0.94 vs. 1.40 ± 1.17, *p* = 0.046). The optimized and dependable NeuroAssist Robotic System improved shoulder and wrist range of motion and functional scores, regardless of the cause of the motor deficit. However, further investigations are necessary to establish its definite role in motor recovery.

## 1. Introduction

Neurological disorders have been recognized as some of the leading causes of disability worldwide [1]. Based on findings by The World Stroke Organization, stroke is the leading cause of long-term disability out of all neurological disorders, with more than 15 million people having an acute cerebrovascular event each year [2]. Motor deficit after stroke can occur in both upper and lower extremities, with variable proportions. The most common deficit in stroke is contralateral upper limb paresis, affecting more than 80% of patients in the acute phase and more than 40% in the chronic phases after stroke [3]. While stroke is the leading cause of upper limb impairment, a series of other neurological disorders, such as multiple sclerosis, cerebral palsy, and spinal cord injuries, lead to loss of function [4].

Upper limb impairment has an important socio-economic and emotional impact on patients, consisting of higher anxiety levels and lower perceived health-related quality of life [5].

The recovery of motor deficits depends on the intensity of the upper limb impairment, with patients with mild to moderate paresis having the best outcomes. It also depends on the time interval between the onset of the neurological event and the moment the rehabilitation procedures began to be applied. The best results were obtained in the first 3 months after function loss [6]. Therefore, adequate and intensive upper limb deficit recovery and rehabilitation are crucial, especially in the early stages after a cerebrovascular, inflammatory, or traumatic event. 

Patients with upper extremity deficiency should practice task-specific training, which consists of repeated, progressive difficulty practice of functional, goal-oriented activities. Strengthening upper extremity muscle exercises can be performed in addition to task-specific training. Constraint-induced movement therapy improves upper limb mobility in patients with baseline ability to control wrist and finger extension compared with usual care. Medical rehabilitation may include mental practice, virtual reality training, neuromuscular electrical stimulation, and somatosensory retraining [7]. To this day, it is still the standard for rehabilitation in most countries. Nevertheless, conventional therapies are time-consuming, labor-intensive, and less cost-effective than newer rehabilitation methods [5].

Robot-assisted therapy (RAT) is a novel approach to rehabilitation that uses different types of robots with the possibility to deliver high dosage and high-intensity training and also allowing better feedback for the patient during the session with less supervision from a therapist [5]. Although the use of robotic devices in rehabilitation therapy has been around since 1960, only in the last 20 years has there been significant growth in the number of newly developed robotic devices [8]. There is a consensus that despite the significant diversity in types of devices evaluated, robot-assisted therapy of the upper limb is generally safe for the patient if used in 30–60 min sessions [9]. Although, until recently, researchers debated the benefit of robot-assisted therapy compared with conventional therapy, a recent review involving 38 randomized trials evaluating the effects of robot-assisted therapy on upper extremity impairment found patients undergoing RAT to have a significantly better Fugl-Meyer scores than those in the conventional therapy groups [10].

Based on their mechanical structures, rehabilitation robots are classified as exoskeleton (EX) and end-effector (EE) types. EX-type robots are connected to the patient at multiple points, their joint axes matching the patient’s joints, whereas EE-type robots have only one point of contact with the patient, usually in the distal region of the affected limb. The force exerted distally in EE-type robots moves more joints simultaneously, making single-joint movement much more difficult than with EX-type robots [10,11].

Robot-assisted therapy has the following objectives: improvement of arm muscle strength and gross motor skills, coordination and stability in the upper limb, smoothness of hand movements and grasping, reduction in spasticity, and improvement of functions in patients with cognitive-motor disorders [2].

The recovery process results can be improved by varying the force applied by the mechanical devices, increasing the movement amplitude, decreasing assistance, and increasing resistance, even with less supervision from therapists [2]. Aphasia, language rehabilitation, and shoulder-hand neuropathic pain may also benefit from robotic-assisted therapy [12,13].

Some recent meta-analyses reported statistically significant but small improvements in motor control and muscle strength of the upper limb (e.g., ~2 Fugl-Meyer points) but found no benefits for upper limb capacity or basic activities of daily living (ADLs) [10,14].

A study by Colombo et al. tested the effectiveness of a 3-week training program with three different robotic devices for shoulder-elbow rehabilitation. They found that all devices tested in this study effectively improved the level of impairment and motor performance. The type of robot device used and the number of training sessions did not seem to influence the final motor outcome [15]. On the same note, another study demonstrated that patients significantly improved their upper limb motor impairment after at least three weeks of robot-assisted training, but subacute patients showed a more significant improvement on the Fugl-Meyer scale than chronic patients [16].

Even though these new approaches to motor rehabilitation using robots seem to bring a new dimension to existing rehabilitation practice, they need to be validated before being used in clinical practice. For clinical use, it is imperative to ascertain that the robotic measures should adapt according to the severity of the paresis. Individuals with moderate to severe upper limb paresis may benefit from robotic therapy that provides a more intensive practice [7].

Most literature studies use rigorous inclusion criteria, mainly focusing on stroke recovery, without considering the vast diversity of neurological causes of motor impairments of the upper limb. Thus, it is necessary to demonstrate the efficiency and the utility of robotic-assisted therapy on a daily basis activity in neurology clinics and in patients suffering from upper limb motor impairment secondary to neurological diseases other than stroke.

In light of these aspects, this study aimed to evaluate the effectiveness of a new robot targeting the shoulder, wrist, and hand motor impairments secondary to stroke and other neurological diseases. After the introduction, the paper’s second section presents the robotic system used for brachial monoparesis rehabilitation, followed by a description of the patients and the medical protocol. The paper’s third section presents the results obtained, followed by a discussion and conclusions.

## 2. Materials and Methods

### 2.1. The Robot

The NeuroAssist Robotic System for upper limb rehabilitation was designed in a modular manner targeting the shoulder (ASPIRE), elbow (ParReEx Elbow), and wrist (ParReEx Wrist) separately. The robotic device uses a unified control system and a user interface that makes simultaneous treatment of 1, 2, or 3 patients possible under the supervision of only one physical therapist. Some of the features of this robotic device were previously explained elsewhere [17,18,19,20,21].

**Figure 1:**
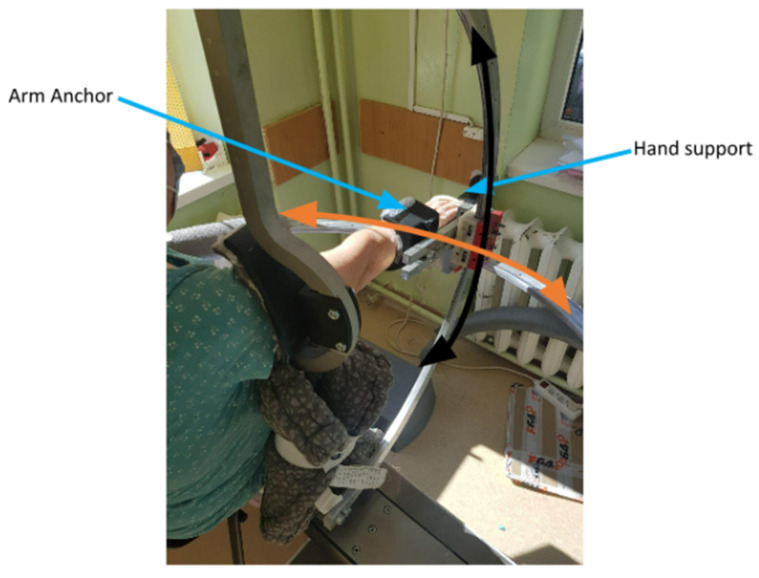
The ASPIRE robotic system.

ASPIRE (see Figure 1) is a spherical robot with two degrees of freedom (DOFs) that targets shoulder rehabilitation and is able to perform flexion/extension (black arrow) and abduction/adduction (orange arrow) motions. The hand is placed in a special support, and the arm is fixed with a Velcro band in the anchor support. 

**Figure 2:**
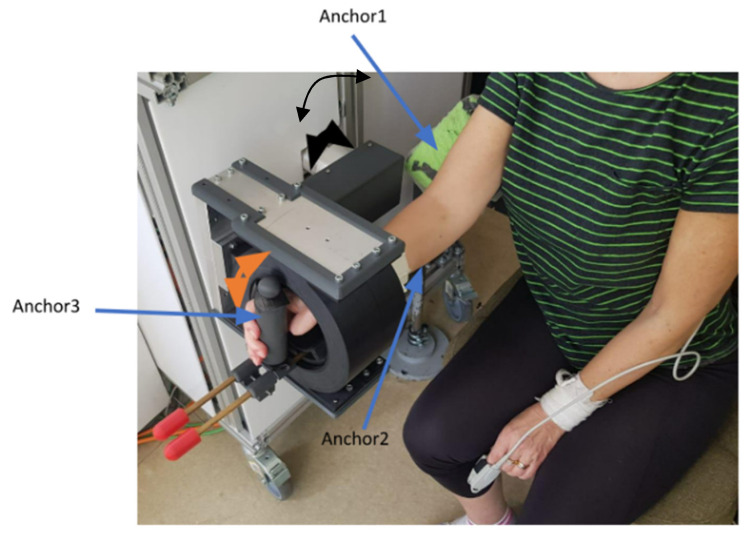
The ParReEx Elbow robotic system.

ParReEx Elbow (see Figure 2) is a parallel robot designed for elbow rehabilitation. The robotic system has two DOFs and can execute the following rehabilitation motions: flexion/extension of the elbow (black arrow) and pronation/supination of the forearm (orange arrow). The arm of the patient is held in place by three anchor points in the elbow rehabilitation module; these points are positioned on the patient: on the upper arm, the forearm, and the hand.

**Figure 3:**
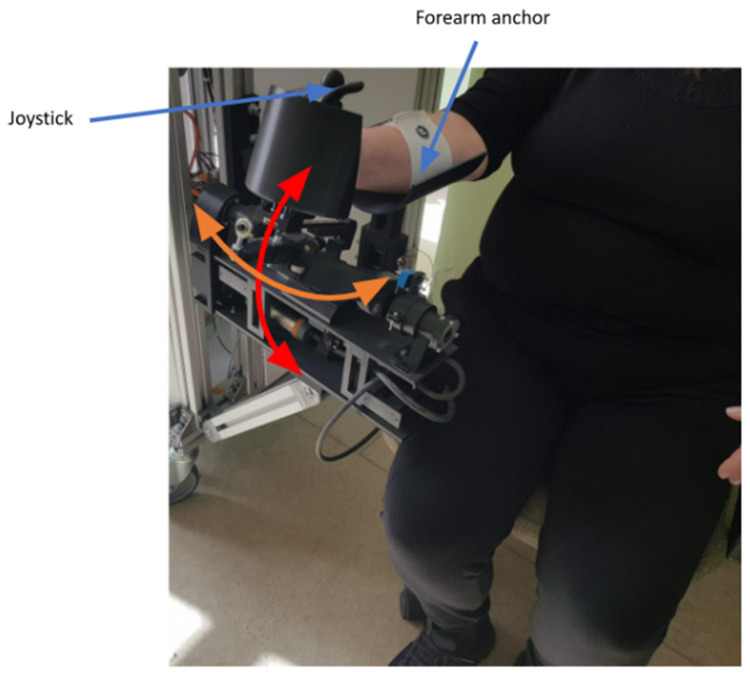
The ParReEx Wrist Robotic System.

ParReEx Wrist (see Figure 3), a parallel robot for wrist rehabilitation, was designed to perform flexion/extension (red arrow) and adduction/abduction (orange arrow) of the wrist. The patient must use their right hand to grasp the wrist rehabilitation module’s joystick (active anchor), which is held in place by a Velcro elastic band.

### 2.2. The Sensors

Internal and external sensor systems measure movement parameters during the medical recovery procedure. The external sensor system monitored the real-time biosignals of the patient and provided base data for the operator to evaluate the patient’s overall health state during the rehabilitation procedure and simultaneously validate the robot mechanisms’ position during the recovery. The internal sensor system was used to monitor the speed during the recovery movement and, simultaneously, the forces occurring in the system.

For the baseline and final assessment of the patient, an external measuring system comprised of goniometers and torsiometers was used. A biomedical, sensory system was used (www.biometricsltd.com) for measuring the motion amplitudes. To measure the motion amplitudes of the wrist, a two-channel goniometer (SG75) with a maximum extension of the flexible element of 75 mm was used. For the elbow joint, a two-channel goniometer (SG110) with a maximum extension of the flexible element of 110 mm was used, and for the shoulder joint also, a two-channel goniometer (SG150) was used with a length of 150 mm for the extension element. Two-channel goniometers were used for measuring the amplitude of flexion/extension/adduction/abduction, and for measuring the pronation/supination amplitude of the forearm, a one-channel torsiometer (Q150) with an e length of the extension element of 170 mm was used.

Dual-axis goniometers measured angles in up to two planes of motion simultaneously. To measure wrist movements, goniometer end blocks were attached to the dorsal surface, one end over the third metacarpal, and the other over the midline of the forearm, with the wrist in a neutral position. The goniometers had two separate output connectors: one measured flexion/extension and the other radial/ulnar deviation.

The goniometers and the torsiometer were connected via connecting wires to a DataLOG unit (Biometrics Ltd., Newport, UK). DataLOG (MWX8) was developed to meet the needs of portable data collection and monitoring research in human performance, sports science, medical research, industrial ergonomics, training laboratories, and educational centers. The DataLOG can be worn on the arm, leg, or waist and incorporates an LCD with color graphics, a joystick, a micro SD card interface, and a real-time connection with Bluetooth^®^ wireless technology to a computer.

Before starting the pilot study and the recruitment of the patients, the entire robotic system was the subject of an extended critical analysis regarding the mechanical structure of the robot, the control system, and the user interface. The aspects regarding the optimizations of the mechanical structure were previously presented [21,22,23]. The control system was optimized for communication protocols and torque monitoring features [19]. The torque monitoring capability was used to implement different human–robot interaction (HRI) strategies [24] and simultaneously assess the patient’s state during the entire rehabilitation procedure. The user interface of the robotic system was redesigned to include new features such as HRI strategies (passive, assistive, active-assistive, and resistive), real-time monitoring of the patient using an external sensor system to monitor the biosignals of the patient (pulse and oxygen saturation level (SpO2)) and provide data regarding the state of the patient to the operator of the robotic system. Another feature of the robotic system is the complex database used and accessed by the system to store data regarding the baseline assessment of the patient, biosignals during the rehabilitation procedure, the amplitudes reached during the procedure, the number of repetitions for each motion, torque evolution during the motion, and the velocity of the motion. The NeuroAssit Robotic System is improved compared with its previous versions in terms of mechanical structure: the finishing of the parts was improved to remove any cause of possible scratches, some transmission elements were replaced to provide better reliability and torque transmission, and some parts were replaced with parts manufactured from more resilient materials. Moreover, in terms of the internal sensors and control system, a faster communication protocol between the PLC (Programmable Logical Computer), the user interface, and torque monitoring capabilities was implemented.

### 2.3. The Patients

The following open-label, interventional clinical study enrolled subacute and chronic patients that suffered from right upper limb impairment (paresis or plegia) of variable neurological causes. 

Patients over 18, regardless of socio-economic background, with right upper limb impairment of variable causes (ischemic or hemorrhagic stroke, multiple sclerosis, and spinal cord injury) who were admitted between April 2022 and August 2022 to the Neurology I Department of the Cluj-Napoca County hospital were enrolled in the study. 

The inclusion criteria were: (a) patients with right upper limb impairment of neurological cause (subacute/chronic ischaemic/hemorrhagic stroke, multiple sclerosis, amyotrophic lateral sclerosis or inflammatory polyneuropathy); (b) the ability to understand and follow instructions; (c) the ability to maintain a sitting position; (d) over 18 years of age; and (e) patients who have given written informed consent for the investigation.

The following exclusion criteria were identified: (a) severe/advanced upper limb arthrosis/arthritis; (b) upper limb trauma that led to severe function loss; (c) sensory or mixed aphasia or cognitive impairment that would diminish the ability to comprehend or perform the investigations, corresponding to a Mini-Mental State Examination (MMSE) score lower than 20 points; (d) refusal or inability to provide written informed consent; and (e) other severe medical problems.

The Ethics Committees of the University of Medicine and Pharmacy of Cluj-Napoca and the County Emergency Hospital of Cluj-Napoca approved this study (approval number AVZ112 from 17 May 2022 for project number PN-III-P2-2.1-PED-2019-3022/546PED/2020). Informed consent was obtained from all study participants, and the study was conducted according to the Helsinki declaration.

### 2.4. The Procedures 

The study included patients of both genders with right upper limb impairment of variable neurological causes. The initial and final assessments were carried out by a team of doctors and physiotherapists, using three different methods applied on day 1 (T1) and day 5 (T2) after the intervention as follows: 

1. Clinical assessment through neurological examination and Barthel Index, ADL scales were performed by a doctor unrelated to the patient. A clinical psychologist also evaluated the patient’s cognitive abilities by performing MMSE.

2. Classical goniometry evaluation for each segment of the upper limb (shoulder, elbow, forearm, wrist) and evaluation with a dynamometer relative to age. A licensed physiotherapist performed these measurements.

3. Electroneuromyography. The motor nerves evaluated in the study were the ulnar and the median, and the sensory ones were the ulnar, radial, and median. The following muscles were evaluated: deltoid, biceps brachialis, flexor carpi radialis, extensor digitorum communis, flexor digitorum superficialis, and abductor pollicis brevis. All the examinations were performed under optimal ambient and body temperature conditions to limit the occurrence of errors. The recorded parameters were: the amplitude of the sensory nerve action potential, sensory nerve conduction velocity, the amplitude of motor nerve action potential, and late responses (F waves). Regarding the electromyographic examination, spontaneous resting state activity, recruitment pattern and duration, amplitude, and phases of motor units were recorded.

### 2.5. The Intervention

The rehabilitation program consisted of a regular number of repetitions: 15 repetitions on days 1 and 2 and 30 repetitions on days 3, 4, and 5. Using the robotic system ASPIRE, the following passive movements were performed: flexion, extension, abduction, and shoulder adduction. With the ParReEx robotic devices, flexion and extension of the elbow, pronation and supination in the forearm, wrist flexion and extension, and ulnar and radial deviation were performed.

The entire rehabilitation program was patient-specific, as the movements were performed slowly to avoid pain, injury, or abnormal activity in the paretic muscles, and the necessary resting phases after each exercise cycle were respected if needed. During the 30 to 45 min therapy sessions, the patients’ heart rates and SpO2 were measured constantly. All the participants received RAT in the same center, on five consecutive days, except weekends, and none received conventional physiotherapy during the intervention or one-month prior. Moreover, all the patients received only their chronic medication (e.g., secondary stroke prevention), and no potential neurotrophic therapies were added.

The robot treatment sessions were administered under the supervision of a doctor and a physiotherapist.

### 2.6. Statistical Analysis

For the descriptive section, means and standard deviations were used as central tendency and dispersion indicators. When comparing pairs of measurements, we used the Wilcoxon test, as the sample is small (*n* = 10). For analyzing the relationships between variables, the correlation matrix (Pearson correlation) was used with the main focus on the intensity of the relationships and not statistical significance, as the sample size (and implicitly the statistical power) is rather small. The data analyses were performed in SPSS 20.

## 3. Results

### 3.1. Clinical and Goniometric Aspects

The study enrolled 10 consecutive patients (2 males) with a mean age of 61.20 (±9.87) years with right upper limb motor deficits caused by different etiologies. Five patients had upper limb deficits due to ischemic stroke, among whom four were in the subacute phase- and none received specific acute treatment (e.g., intravenous thrombolysis). Two patients had a hemorrhagic stroke in the chronic phase, two subjects suffered from secondary progressive multiple sclerosis, and one had suffered a traumatic spinal cord injury one year before enrolment in the study. 

There were significant differences between baseline and end-point clinical parameters regarding the Barthel Index (53.00 ± 37.72 at T1 vs. 60.50 ± 36.39 at T2, *p* = 0.016) and Activities of Daily Living Index (4.70 ± 3.43 vs. 5.50 ± 3.80, *p* = 0.038). The evaluated goniometric parameters were also improved: shoulder flexion (70.00 ± 56.61 at T1 vs. 80.00 ± 63.59 at T2, *p* = 0.026); wrist flexion (34.00 ± 28.75 vs. 42.50 ± 33.7, *p* = 0.042); wrist extension (30.00 ± 22.97 vs. 41.00 ± 30.62, *p* = 0.042); ulnar deviation (23.50 ± 19.44 vs. 33.50 ± 24.15, *p* = 0.027); and radial deviation (17.50 ± 18.14 vs. 27.00 ± 24.85, *p* = 0.027). There were no significant statistical differences regarding the distal muscle force of the upper limb measured with the dynamometer nor of the muscle segmental strength (Table 1).

Significant correlations were observed between the Barthel Index and all classical goniometric parameters analyzed both before and after the use of the NeuroAssist Robot (see Table 2 and Table 3).

### 3.2. Nerve Conduction Studies and Electromyography

As mentioned earlier, all the patients underwent motor and sensory nerve conduction studies of the nerves, but no significant differences were observed between the T1 and T2 measurements (see Table 4). There was a significant difference in the activation, examined via electromyography of the extensor digitorum communis muscle (1.00 ± 0.94 vs. 1.40 ± 1.17, *p* = 0.046), and also at the level of the deltoid, biceps, and flexor carpi radialis muscles, but these differences did not reach the level of significance (see Table 5).

### 3.3. Range of Motion Measurements

Table 6 shows the motion amplitudes and speeds during the rehabilitation sessions. The motion characteristics were recorded by monitoring the encoders of servomotors. The correlation between the recorded amplitudes and the motion amplitudes on the experimental subject was validated during laboratory tests. Significant differences were observed in the parameters regarding wrist flexion/extension (48.00 ± 15.846 vs. 55.00 ± 13.744, *p* = 0.005/42.50 ± 10.34 vs. 48.50 ± 11.068, *p* = 0.005), abduction/adduction amplitudes (22.0 ± 7.528 vs. 26.00 ± 6.146, *p* = 0.011/31.00 ± 9.944 vs. 35.00 ± 5.774, *p* = 0.020) at the same speed (Wrist flexion-extension/abduction-adduction—30.00 degrees/second) but at increased intensity (15 repetitions/minute in the first session and 30 repetitions/minute in the last one). A similar significant pattern was obtained for the shoulder flexion and shoulder abduction (see Table 6.) 

The range of motion of shoulder, wrist, and elbow joints was measured electronically with the above-mentioned sensors attached to the robot. The range of motion differed significantly between T1 and T2 for shoulder flexion (68.46 ± 55.37 vs. 78.24 ± 62.20, *p* = 0.026), wrist flexion/extension (33.25 ± 28.12 vs. 41.57 ± 33.03, *p* = 0.042/29.34 ± 22.47 vs. 40.10 ± 29.95, *p* = 0.042), and ulnar/radial deviation (22.98 ± 19.02 vs. 32.76 ± 23.63, *p* = 0.027/17.12 ± 17.74 vs. 26.41 ± 24.31, *p* = 0.026) (Table 7).

Universally, the goniometric measurements showed significantly strong intensity Pearson’s correlation coefficients with the electronic measurements regarding all measured joint movements (shoulder flexion/ abduction, elbow flexion/extension, pronation/supination, and ulnar/ radial deviation) both at T1 (see Table 8) and after the last therapeutic session (see Table 9). Statistical significance was at *p* < 0.01.

### 3.4. Vitals during Therapy Sessions

**Figure 4:**
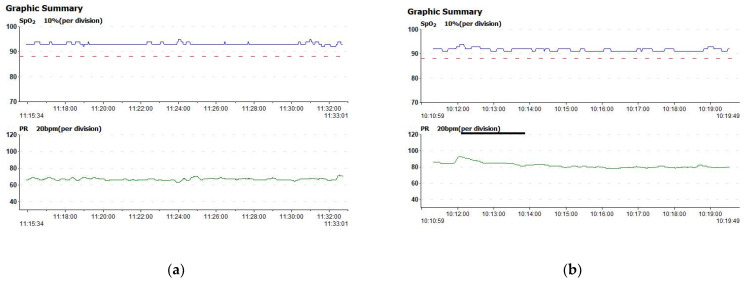
Examples of patient oxygen saturation and heart rate during the Aspire robot sessions in (**a**) the first session and (**b**) the last session.

Each patient’s heart rate variability and oxygen saturation were monitored using an integrated finger pulse oximeter. The SpO2 and heart rate remained stable during each session; an example is given in Figure 4a,b. 

## 4. Discussion

Given the fact that stroke is the leading cause of disability worldwide and that the most common deficit in stroke is contralateral upper limb paresis, affecting more than 80% of patients in the acute phase and more than 40% in the chronic phases after stroke [5], it is expected that many studies focus on analyzing RAT in the recovery of the upper limb; thus, this study also enrolled mainly stroke survivors. In the present day, as stroke is a socio-economic burden and there are many therapeutic possibilities for dealing with motor impairments and post-stroke disabilities, most current studies focus on developing improved rehabilitation therapies for those stroke survivors. A few studies focus on RAT in multiple sclerosis [25,26] or spinal cord injuries [27,28,29]. However, this study, even if small, included patients with upper limb impairments caused by neurological disorders other than stroke. 

Although the use of robotic devices in rehabilitation therapy has been around for the last half of a century, only in the last 20 years has there been significant growth in the number of newly developed robotic devices, for example, ARMin, MIME, MIT-MANUS, Armeo-Spring, GENTLE/s and NeReBot [8,30,31,32,33,34]. These devices provide participants with repetitive and quantifiable physical training and different types of sensory-motor feedback [35]. Most of the robotic rehabilitation devices developed so far target one to two articulations (e.g., shoulder and elbow MIT-MANUS, ARMin, Act [31]) and comprise no more than two degrees of freedom (DOFs). A few robotic devices have been developed to target three DOFs, e.g., GENTLE/s [30,32] and NeReBot [34]. 

The NeuroAssist Robotic System is composed of three modular robotic structures targeting the shoulder, elbow, and wrist, with two DOFs for each of them, and it was developed to deliver ergonomic movements to patients. These results proved that the intervention applied with this modular robot improved the range of motion as measured with a universal goniometer at the level of shoulder flexion, wrist flexion, and extension, as well as ulnar deviation and radial deviation (Table 1). A significant improvement in the Barthel Index and ADLs was observed after the intervention, reflecting a higher level of independence after being released from hospital. Other studies analyzing the effect of RAT on stroke demonstrated improvement in the Barthel index or the Fugh-Meyer Scale [30,36,37,38]. 

In a single-blind, randomized, controlled trial conducted by Dehem and collaborators [39] on 45 patients divided into two groups, one for conventional therapy and one for RAT combined with CT, concluded that for the same duration of rehabilitation, 9 weeks, the group that received combined therapy had better outcomes with a significantly better improvement of gross motor dexterity in the upper limb than the group that received conventional therapy alone [39]. The study only enrolled patients in the acute and subacute phases of stroke (less than 6 months post-event) and used an end-effector type of robotic device so that the following conclusions can be drawn: robotic-assisted therapy can partially substitute conventional therapy with a clear improvement of gross manual dexterity in the upper limb and patient social participation. 

The current study included patients in subacute and chronic stages that improved after RAT with NeuroAssist System, and these positive effects are more likely due to the interventions rather than to spontaneous recovery, which is more common in acute and subacute stages. Furthermore, in the present pilot study, the patients did not receive combined CT and RAT therapy, but using NeuroAssist Systems together with CT would have produced positive and more encouraging results.

Another study [40] comparing the efficiency of combined robotic-assisted and conventional therapy versus CT alone was a randomized controlled trial conducted between 2016 and 2018 by Budhota and his collaborators and enrolled 44 patients divided into two equal groups. Both groups benefited from 90-min sessions three times a week over 6 weeks. The RAT group had a 60-min rehabilitation session performed with H-Man, a two-degree of freedom compact robot, followed by 30 min of conventional therapy, while the CT group benefitted from 90 min of conventional therapy. Both groups had significant retained improvement by the end of the 6 weeks on all clinical scales, but no statistically significant differences were found between the two groups. The study concluded that “Time matched combinatory robotic therapy that integrates robotic aided therapy and therapist supervised therapy in a 2:1 ratio using H-Man was safe, efficacious, and acceptable in a supervised manner”, with the stroke participants who underwent combined rehabilitation having improvements in motor function in the upper limb comparable with those of participants who underwent conventional therapy alone [40].

There is a consensus that despite the significant diversity in types of devices evaluated, robot assisted therapy of the upper limb is generally safe for the patient if used in 30–60 min sessions [9], and the subjects involved in this study received five sessions of 30 to 45 min each. Most of the studies applied different amounts of RAT for three sessions/week for 3 to 4 weeks and some showed improvement, but there is a lack of recommendations regarding the amount of RAT needed to observe clinical effects.

These results suggest that RAT with NeuroAssist Robot is clinically efficient, even in shorter therapy programs, as the patients underwent five sessions but still experienced an improvement in analyzed upper limb ROMs and Barthel and ADL indexes. Compared with several studies [17,18] that included patients with upper limb motor deficits caused by stroke or extrapyramidal disorders, this pilot study included only patients with motor impairment caused by the lesion of the upper neuron, a pyramidal disorder, making the current analyzed group more homogenous from this point of view. Moreover, the amount of RAT and the evaluation protocol in this pilot study was different, and further comparison to the earlier studies [17,18] is inappropriate.

Electromyography can identify the differences in muscle activation early in the course of neurological diseases or after therapeutical interventions as muscle activity is the first to change [41]. An improvement in the activation of extensor digitorum communis and deltoid muscle was obtained; thus, these findings consolidate the improvement of the clinical parameters. Other studies used surface EMG to monitor muscle activity during RAT sessions and to control the amount of RAT given to each patient [42]. In the present study, we used conventional needle EMG to assess muscle activity before and after the intervention. The significant differences observed in the muscle activation suggest that the benefits of using the NeuroAssist Robot last beyond the last session and that the repetitive movements applied by the robot stimulate motor recovery. 

Furthermore, patients improved their shoulder flexion, wrist flexion/extension, and radial/ulnar deviation range of motion, as measured with a universal goniometer and electronic sensors. 

The NeuroAssist Robotic System is improved compared with its previous versions in terms of mechanical structure, control system, and internal sensors, with a faster communication protocol between the PLC and the user interface and torque monitoring capabilities.

The new features implemented within the NeuroAssist Robotic System refer to the interaction between the robotic system, patient, and operator by using different human–robot interaction modes (passive, assistive, active-assistive, and resistive). An internal sensor system is used to check the position of the elements of the robot during the procedure, and at the same time, to monitor the forces/torques from the system. In addition, an external sensor system checks the state of the patient during the procedure by monitoring the pulse, oxygen saturation level, and range of motion. All the above features were recorded during the rehabilitation procedure and stored using a complex database accessed by the robotic system and used to assess the state and the evolution of the patient simultaneously with the robotic system. The robotic system used dependable external sensors to assess the patient’s state during the rehabilitation procedure. There were significant strong correlations between ROM measured classically and with the external sensors, indicating that the external sensors are reliable and feasible for use in clinical trials or daily practice.

Furthermore, the integrated HRI modalities increased patient comfort during robot-assisted training as all the patients completed the intervention.

In MS, adaptive robot training may be an effective strategy to enhance upper limb kinematics and functional abilities [25]. In a pilot study, RAT with The Armeo Spring proved efficacious in improving functional capacity [26]. The current study enrolled two patients with secondary progressive MS that improved their range of motion and functional scores, indicating that the NeuroAssit Robotic System is also feasible for upper limb rehabilitation in MS. 

During each session, the patients’ SpO2 and heart rate remained relatively stable, proving the safeness of the NeuroAssist robotic device for cardiovascular integrity, as many patients refuse to engage in neurorehabilitation programs as they feel fatigued [43].

### Study LIMITS and Future Endeavors

The limitations of this study are the small sample size and the lack of a control group, but it has the advantage of EMG investigation in addition to the clinical and goniometric evaluations. Another limitation is the lack of use of the Fugl-Meyer scale [1]; this was not used due to the heterogeneity of etiology in the upper limb motor deficits. Besides stroke, some of the patients enrolled in this study suffered from multiple sclerosis or spinal cord injuries, and the Fugl-Meyer motor scale is a highly recommended clinical and research tool for assessing changes in motor disability after stroke [44]. 

## 5. Conclusions

The modular robotic system proved effective in the evaluated parameters, regardless of the cause of the motor deficit: stroke, traumatic spinal cord disease, or multiple sclerosis.

The optimized NeuroAssist Robotic System proved efficient in rehabilitating the upper limb motor deficits, significantly improving the range of motion of the shoulder flexion and wrist flexion and extension, forearm radial/ulnar deviation, and functional scales. 

The newly incorporated human–robot interaction interfaces to NeuroAssist, together with the external sensors, enhance the control of the robotic system and its reliability. The positive results of this pilot study are encouraging, but further investigations are necessary to establish its definite role in motor recovery. Future studies should include a larger number of subjects and compare the NeuroAssist interventions to conventional physiotherapy, as the current sample size is small. Moreover, including patients with motor impairments caused by the lesion of the peripheral motor neuron (e.g., brachial plexopathy) warrants exploration in the future.

## Figures and Tables

**Table 1:** Clinical and goniometric features of the group before (T1) and after (T2) RAT (Wilcoxon test). Results are represented as mean (SD).

	T1	T2	*Z*	*p*
Barthel Index (points)	53.00 (37.72)	60.50 (36.39)	−2.41	0.016
ADL (points)	4.70 (3.43)	5.50 (3.80)	−2.07	0.038
Dynamometer (kg)	8.33 (8.37)	9.89 (10.06)	−1.57	0.116
Muscle segment strength (Scale)	2.70 (1.61)	3.30 (0.91)	−1.85	0.063
Shoulder abduction (degrees)	54.00 (47.71)	58.50 (52.33)	−1.34	0.180
Shoulder flexion (degrees)	70.00 (56.61)	80.00 (63.59)	−2.20	0.026
Elbow flexion (degrees)	105.50 (59.18)	120.50 (55.35)	−1.82	0.068
Wrist flexion (degrees)	34.00 (28.75)	42.50 (33.76)	−2.03	0.042
Wrist extension (degrees)	30.00 (22.97)	41.00 (30.62)	−2.03	0.042
Ulnar deviation (degrees)	23.50 (19.44)	33.50 (24.15)	−2.21	0.027
Radial deviation (degrees)	17.50 (18.14)	27.00 (24.85)	−2.20	0.027
Pronation (degrees)	52.50 (45.41)	53.50 (46.07)	−1.00	0.317
Supination (degrees)	47.50 (44.04)	54.50 (41.79)	−1.60	1.109

**Table 2:** Correlation matrix (Pearson correlation coefficient) at T1 between the clinical parameters and classical goniometric findings (* Correlation is significant at the 0.01 level (2-tailed), ** Correlation is significant at the 0.05 level (2-tailed)).

Variables	1	2	3	4	5	6	7	8	9	10	11	12	13
1. Barthel Index	-												
2. ADL Index	0.98 **	-											
3. Muscle strength	0.78 **	0.80 **	-										
4. Shoulder abduction	0.84 **	0.86 **	0.91 **	-									
5. Shoulder flexion	0.92 **	0.94 **	0.91 **	0.95 **	-								
6. Elbow flexion	0.91 **	0.91 **	0.83 **	0.88 **	0.92 **	-							
7. Wrist flexion	0.78 **	0.74 *	0.70 *	0.72 *	0.78 **	0.81 **	-						
8. Wrist extension	0.85 **	0.83 **	0.65 *	0.62	0.78 **	0.83 **	0.78 **	-					
9. Ulnar deviation	0.78 **	0.79 **	0.78 **	0.76 **	0.83 **	0.88 **	0.80 **	0.91 **	-				
10. Radial deviation	0.77 **	0.77 **	0.82 **	0.88 **	0.89 **	0.75 *	0.82 **	0.57	0.65 *	-			
11. Pronation	0.85 **	0.85 **	0.91 **	0.98 **	0.93 **	0.89 **	0.71 *	0.62	0.75 *	0.84 **	-		
12. Supination	0.75 *	0.75 *	0.90 **	0.95 **	0.87 **	0.88 **	0.75 *	0.62	0.82 **	0.81 **	0.96 **		
13. Dynamometer	0.72 *	0.74 *	0.76 **	0.85 **	0.84 **	0.75 *	0.68 *	0.72 *	0.85 **	0.80 **	0.77 **	0.81 **	-

**Table 3:** Correlation matrix (Pearson correlation coefficient) at T2 between the clinical parameters and classical goniometric findings (* Correlation is significant at the 0.01 level (2-tailed), ** Correlation is significant at the 0.05 level (2-tailed)).

Variables	1	2	3	4	5	6	7	8	9	10	11	12	13
1. Barthel	1												
2. ADL	0.98 **	1											
3. FMS	0.59	0.66 *	1										
4. Shoulder abduction	0.86 **	0.90 **	0.84 **	1									
5. Shoulder flexion	0.93 **	0.95 **	0.80 **	0.95 **	1								
6. Elbow flexion	0.85 **	0.87 **	0.51	0.75 *	0.82 **	1							
7. Wrist flexion	0.87 **	0.89 **	0.70 *	0.86 **	0.89 **	0.80 **	1						
8. Wrist extension	0.88 **	0.86 **	0.57	0.78 **	0.82 **	0.85 **	0.93 **	1					
9. Ulnar deviation	0.92 **	0.91 **	0.54	0.81 **	0.85 **	0.87 **	0.95 **	0.98 **	1				
10. Radial deviation	0.78 **	0.78 **	0.64 *	0.81 **	0.79 **	0.69 *	0.90 **	0.78 **	0.82 **	1			
11. Pronation	0.89 **	0.90 **	0.76*	0.96 **	0.93 **	0.76 **	0.81 **	0.77 **	0.80 **	0.81 **	1		
12. Supination	0.94 **	0.94 **	0.72 *	0.95 **	0.94 **	0.85 **	0.90 **	0.89 **	0.91 **	0.85 **	0.97 **	1	
13. Dynamometer	0.75 *	0.82 **	0.73 *	0.88 **	0.82 **	0.64 *	0.90 **	0.73 *	0.79 **	0.87 **	0.79 **	0.81 **	

**Table 4:** Nerve conduction studies before (T1) and after (T2) RAT (Wilcoxon test). Results are represented as mean (SD). ENoG—electroneurography, aSNAP—amplitude of sensory action potential, mNCV—motor nerve velocity, aCMAP—amplitude of compound muscle action potential.

ENoG Parameters	T1	T2	*Z*	*p*
aCMAP median (mV)	6.40 (2.34)	6.95 (2.32)	−1.21	0.225
mVCM median (m/s)	58.55 (8.79)	58.66 (8.03)	−1.21	0.500
F waves median (ms)	23.63 (2.64)	23.03 (2.93)	−0.67	0.500
aCMAP ulnar (mV)	6.75 (2.24)	6.45 (2.38)	−1.57	0.116
mVCM ulnar (m/s)	67.45 (9.67)	62.95 (9.94)	−1.48	0.138
F waves ulnar (ms)	24.85 (3.52)	25.27 (3.27)	−0.13	0.893
aSNAP median (µV)	16.34 (15.04)	13.71 (11.97)	−0.40	0.686
aSNAP ulnar (µV)	16.44 (18.12)	15.41 (18.44)	−0.67	0.500
aSNAP radial (µV)	18.82 (5.46)	16.43 (6.20)	−1.36	0.173

**Table 5:** Electromyographic parameters before (T1) and after (T2) RAT ((Wilcoxon test). Results are represented as mean (SD). APB—abductor policis brevis muscle, FCR—flexor carpi radialis, EDC—extensor digitorum communis, FDS—flexor digitorum superficialis, FIB—fibrillation potentials, PSW—positive sharp waves, MAUP—motor unit action potential, amp—amplitude.

	T1	T2	*Z*	*p*
ABP FIB	0.40 (0.69)	0.90 (1.10)	−1.63	0.102
ABP PSW	0.00 (0.00)	0.30 (0.48)	−1.73	0.083
ABP activation	1.00 (0.94)	1.10 (1.10)	−1.00	0.317
ABP MAUP amp	452.50 (267.62)	531.83 (249.27)	−0.73	0.461
ABP MAUP duration	15.15 (11.36)	13.11 (6.08)	−0.67	0.500
ABP polyphasic	41.67 (40.82)	29.67 (35.01)	−0.73	0.465
ABP recruitment	1.10 (0.99)	1.20 (1.13)	−1.00	0.317
FCR FIB	0.10 (0.31)	0.20 (0.63)	−0.44	0.655
FCR PSW	0.00 (0.00)	0.00 (0.00)	0.00	1.000
FCR activation	1.00 (0.94)	1.30 (0.94)	−1.73	0.083
FCR MAUP amp	498.67 (334.72)	531.71 (398.33)	−0.73	0.465
FCR MAUP duration	12.53 (3.59)	13.97 (6.99)	−0.36	0.715
FCR polyphasic	37.00 (26.53)	40.14 (30.85)	−0.36	0.715
FCR recruitment	1.10 (0.99)	1.30 (0.949)	−1.41	0.157
EDC FIB	0.30 (0.67)	0.40 (0.69)	−0.57	0.564
EDC PSW	0.10 (0.31)	0.10 (0.31)	0.00	1.000
EDC activation	1.00 (0.94)	1.40 (1.17)	−2.00	0.046
EDC MAUP amp	665.00 (163.24)	822.14 (339.40)	−1.21	0.225
EDC MAUP duration	19.17 (6.61)	19.88 (8.00)	−0.40	0.684
EDC polyphasic	63.29 (35.44)	65.86 (36.88)	−1.13	0.257
EDC recruitment	1.10 (0.99)	1.60 (1.35)	−1.89	0.059
Biceps FIB	0.20 (0.63)	0.40 (0.69)	−0.55	0.577
Biceps PSW	0.00 (0.00)	0.10 (0.31)	−1.00	0.317
Biceps activation	1.30 (0.82)	1.60 (0.96)	−1.73	0.083
Biceps MAUP amp	524.13 (221.94)	510.63 (230.02)	−0.94	0.345
Biceps MAUP duration	15.05 (7.52)	13.20 (5.99)	−1.78	0.075
Biceps polyphasic	39.00 (35.78)	25.13 (35.58)	−1.36	0.173
Biceps recruitment	1.30 (0.82)	1.50 (0.97)	−1.41	0.157
Deltoid FIB	0.30 (0.67)	0.60 (0.84)	−1.34	0.180
Deltoid PSW	0.10 (0.31)	0.20 (0.42)	−1.00	0.317
Deltoid activation	1.30 (0.94)	1.60 (0.96)	−1.73	0.083
Deltoid MAUP amp	584.38 (166.29)	587.67 (337.83)	−0.52	0.600
Deltoid MAUP duration	18.97 (6.06)	13.144 (3.88)	−1.99	0.046
Deltoid polyphasic	60.25 (33.20)	39.56 (23.06)	−1.75	0.080
Deltoid recruitment	1.50 (1.08)	1.70 (1.05)	−1.41	0.157
FDS FIB	0.00 (0.00)	0.00 (0.00)	0.00	1.000
FDS PSW	0.00 (0.00)	0.00 (0.00)	0.00	1.000
FDS activation	1.00 (1.054)	1.20 (1.13)	−1.41	0.157
FDS MAUP amp	597.33 (231.20)	630.50 (336.98)	−0.40	0.686
FDS MAUP duration	14.50 (4.62)	13.783 (5.41)	−0.27	0.786
FDS polyphasic	41.33 (35.27)	48.17 (31.09)	−0.13	0.893
FDS recruitment	1.10 (1.10)	1.30 (1.16)	−1.41	0.157

**Table 6:** Motion parameters of the RAT parameters before (T1) and after (T2) RAT ((Wilcoxon test). Results are represented as mean (SD).

	T1	T2	*Z*	*p*
Wrist flexion amplitude (degrees)	48.00 (15.846)	55.00 (13.744)	−2.80	0.005
Wrist extension amplitude (degrees)	42.50 (10.341)	48.50 (11.068)	−2.80	0.005
Wrist flexion-extension speed (degrees/second)	30.00 (0.00)	30.00 (0.00)	0.00	1.000
Wrist flexion-extension repetitions/minute	15.00 (0.00)	30.00 (0.00)	−3.16	0.002
Wrist abduction amplitude (degrees)	22.00 (7.528)	26.00 (6.146)	−2.53	0.011
Wrist adduction amplitude (degrees)	31.00 (9.944)	35.00 (5.774)	−2.33	0.020
Wrist abduction-adduction speed (degrees/second)	30.00 (0.00)	30.00 (0.00)	0.00	1.000
Wrist abduction-adduction repetitions/minute	15.00 (0.00)	30.00 (0.00)	−3.16	0.002
Elbow flexion amplitude (degrees)	73.50 (2.41)	73.50 (2.41)	0.00	1.000
Elbow extension amplitude (degrees)	13.50 (2.41)	13.50 (2.41)	0.00	1.000
Elbow flexion-extension speed (degrees/second)	10.00 (0.00)	10.00 (0.00)	0.00	1.000
Elbow flexion-extension repetitions/minute	15.00 (0.00)	30.00 (0.00)	−3.16	0.002
Elbow pronation amplitude (degrees)	60.00 (0.00)	60.00 (0.00)	0.00	1.000
Elbow supination amplitude (degrees)	45.00 (0.00)	45.00 (0.00)	0.00	1.000
Elbow pronation/supination speed (degrees/second)	15.00 (0.00)	15.00 (0.00)	0.00	1.000
Elbow pronation/supination repetitions/minute	15.00 (0.00)	30.00 (0.00)	−3.16	0.002
Shoulder flexion amplitude (degrees)	61.00 (4.59)	62.50 (4.85)	−1.73	0.083
Shoulder extension amplitude (degrees)	0.00 (0.00)	0.00 (0.00)	0.00	1.000
Shoulder flexion-extension speed (degrees/second)	10.00 (0.00)	10.00 (0.00)	0.00	1.000
Shoulder flexion-extension repetitions/minute	15.00 (0.00)	30.00 (0.00)	−3.16	0.002
Shoulder abduction amplitude (degrees)	29.00 (16.63)	33.50 (16.50)	−2.06	0.039
Shoulder adduction amplitude (degrees)	29.00 (3.16)	30.00 (0.00)	1.00	0.317
Shoulder abduction-adduction speed (degrees /second)	20.00 (0.00)	20.00 (0.00)	0.00	1.000
Shoulder abduction-adduction repetitions/minute	15.00 (0.00)	30.00 (0.00)	−3.16	0.002

**Table 7:** Comparison between baseline and final assessments using external measuring systems before (T1) and after (T2) RAT. (Wilcoxon test). Results are represented as mean (SD).

	T1	T2	*Z*	*p*
Shoulder abduction	52.81 (46.66)	57.21 (51.19)	−1.34	0.180
Shoulder flexion	68.46 (55.37)	78.24 (62.20)	−2.22	0.026
Elbow flexion	103.18 (55.37)	117.85 (54.13)	−1.82	0.068
Wrist flexion	33.25 (28.12)	41.57 (33.03)	−2.03	0.042
Wrist extension	29.34 (22.47)	40.10 (29.95)	−2.03	0.042
Ulnar deviation	22.98 (19.02)	32.76 (23.63)	−2.21	0.027
Radial deviation	17.12 (17.74)	26.41 (24.31)	−0.20	0.026
Pronation	51.35 (44.42)	52.32 (45.06)	−1.00	0.317
Supination	46.46 (43.08)	53.30 (40.88)	−1.60	0.109

**Table 8:** Correlation matrix (Pearson correlation coefficient) at T1 between the sensor measurements and classical goniometric findings (* Correlation is significant at the 0.01 level).

Goniometer	Shoulder Abduction	Shoulder Flexion	Elbow Flexion	Wrist Flexion	Wrist Extension	Ulnar Deviation	Radial Deviation	Pronation	Supination
Sensors									
Shoulder abduction	1								
Shoulder flexion	0.950 *	1							
Elbow flexion	0.885 *	0.922 *	1						
Wrist flexion	0.722 *	0.783 *	0.817 *	1					
Wrist extension	0.628 *	0.784 *	0.832 *	0.782 *	1				
Ulnar deviation	0.768 *	0.833 *	0.887 *	0.802 *	0.914 *	1			
Radial deviation	0.889 *	0.898 *	0.759 *	0.820 *	0.573 *	0.658 *	1		
Pronation	0.984 *	0.933 *	0.891 *	0.715 *	0.623 *	0.750 *	0.845 *	1	
Supination	0.952 *	0.878 *	0.880 *	0.759 *	0.620 *	0.822 *	0.812 *	0.960 *	1

**Table 9:** Correlation matrix (Pearson correlation coefficient) at T2 between the sensor measurements and classical goniometric findings (* Correlation is significant at the 0.01 level).

Goniometer	Shoulder Abduction	Shoulder Flexion	Elbow Flexion	Wrist Flexion	Wrist Extension	Ulnar Deviation	Radial Deviation	Pronation	Supination
Sensors									
Shoulder abduction	1								
Shoulder flexion	0.954 *	1							
Elbow flexion	0.758 *	0.825 *	1						
Wrist flexion	0.867 *	0.891 *	0.809 *	1					
Wrist extension	0.788 *	0.827 *	0.850 *	0.935 *	1				
Ulnar deviation	0.811 *	0.852 *	0.871 *	0.952 *	0.982 *	1			
Radial deviation	0.812 *	0.794 *	0.694 *	0.900 *	0.785 *	0.829 *	1		
Pronation	0.968 *	0.932 *	0.767 *	0.810 *	0.777 *	0.801 *	0.816 *	1	
Supination	0.956 *	0.949 *	0.851 *	0.902 *	0.899 *	0.915 *	0.854 *	0.972 *	1

## Data Availability

The data presented in this study are available upon request, due to ethical restrictions.
